# Overview of the Typhoid Conjugate Vaccine Pipeline: Current Status and Future Plans

**DOI:** 10.1093/cid/ciy884

**Published:** 2019-02-15

**Authors:** Sushant Sahastrabuddhe, Tarun Saluja

**Affiliations:** International Vaccine Institute, Seoul, Republic of Korea

**Keywords:** *Salmonella* Typhi, typhoid conjugate vaccines, immunogenicity, safety

## Abstract

Typhoid fever remains a common and serious disease in populations that live in low- and middle-income countries. Treatment usually consists of antibiotics, but problems with drug-resistant strains have been increasing in endemic countries, making treatment prolonged and costly. Improved sanitation and food hygiene have been effective in controlling the disease in the industrialized world, but these steps are associated with socioeconomic progress that has been slow in most of the affected areas. Therefore, vaccination is an effective way to prevent the disease for the short to medium term. Oral typhoid vaccine and Vi polysaccharide typhoid vaccine (Vi polysaccharide) have been available for many years, yet a large population, in particular infants and children aged <2 years, remains at higher risk. Recently, with the availability of Vi polysaccharide–based conjugate vaccines and funding to support vaccination from the Gavi alliance, there is great momentum for typhoid prevention efforts. Supply of the vaccine will be critical, and there are multiple efforts to make new typhoid vaccines accessible and available to populations that desperately need them.

## DISEASE AND PATHOGEN

Typhoid (enteric) fever is an important cause of morbidity and mortality. It is caused by infection with *Salmonella enterica serovar* Typhi (*S*. Typhi), a gram-negative bacterium that invades the body via the small intestines and colonizes macrophages in the reticuloendothelial system, where it is shed into the bloodstream [1, 2]. Symptoms of the resulting disease typically include prolonged fever, frontal headache, malaise, and marked loss of appetite, sometimes accompanied by abdominal pain, nausea, and, in severe cases, intestinal perforation and neurological complications [3]. Symptoms typically subside in 7–21 days, but mortality is estimated at 1%–5% of hospitalized patients [4–6]. In a small percentage of cases, the bacteria may also colonize the gallbladder, leading to a chronic carrier state [3]. 

Between 11.9 and 26.9 million cases of typhoid fever occur each year in low- and middle-income countries [7]. Most cases can be treated effectively with antibiotics. However, antibiotic resistance is a challenge for effective treatment of typhoid, and treatment is likely to become increasingly problematic with the spread of multidrug-resistant strains [8]. Vaccination against typhoid has proven to be an effective preventive intervention, especially when coupled with hand-washing, treatment of household water, and provision of adequate sanitation and other preventive measures [9].

In 2008, the World Health Organization (WHO) recommended vaccination of all children in areas where the disease is common and of those at high risk [10]. At a meeting held on 17–19 October 2017 in Geneva, Switzerland, the Strategic Advisory Group of Experts on Immunization recommended the introduction of typhoid conjugate vaccine (TCV) for infants and children aged >6 months as a single dose in typhoid-endemic countries [11]. Typhoid vaccines are on the WHO’s list of essential medicines, which are the most effective and safe medicines needed within a health system [12].

## VACCINE CANDIDATE PIPELINE AND STATUS

### Whole Cell *S*. Typhi Vaccine

Almroth Edward Wright, Richard Pfeiffer, and Wilhelm Kolle developed the first typhoid vaccine in 1896 [13]. It was a heat-killed, phenol-preserved, and acetone-killed lyophilized injectable whole-cell *S*. Typhi vaccine that was used in England and Germany. The efficacy of this vaccine was assessed in a trial in 1960 in Yugoslavia, Russia, Poland, and Guyana. Although licensed in few countries, this vaccine is no longer used due to its side effects.

### The Live Attenuated Ty21a

Due to limitations of the killed whole-cell vaccine, there was a need to develop a more competent vaccine candidate. With the knowledge that a live attenuated strain elicits more immune response, attenuated *Salmonella* strains were considered for vaccine development. Ty21a, the first live oral attenuated *Salmonella* vaccine (sold as Vivotif by Berna Biotech, then Crucell and now PaxVax), was developed in Switzerland by chemical mutagenesis of wild-type *S*. Typhi strain Ty2 [14, 15]. This strain lacks both the functional galactose-epimerase gene and the Vi antigen and is highly attenuated. This vaccine is available as a liquid and as an enteric-coated capsule. Various clinical trials have reported efficacy up to 67% for more than 7 years. Despite an adequate immune response and efficacy against typhoid fever, Ty21a has some drawbacks. To obtain sufficient immunity, high numbers (10^9^) of bacteria are required for the oral dose; its use is recommended only for children aged >5 to 6 years (because of capsule availability). Because this vaccine is highly acid-labile, stomach acidity has to be neutralized or bypassed when Ty21a is fed orally [16]. Ty21a vaccine is not a WHO-prequalified vaccine [17].

### Vi Polysaccharide Vaccine

The Vi capsular polysaccharide vaccine, a subunit vaccine, was first licensed in the United States in 1994 and is made from the purified Vi capsular polysaccharide from the Ty2 *Salmonella* Typhi strain. As for other polysaccharide vaccines, the Vi vaccine is not effective in children aged <2 years. The vaccine is moderately immunogenic (approximately 65%) and requires repeat dosing every 3 years [18, 19]. Typhim Vi (manufactured by Sanofi Pasteur) was WHO prequalified in 2011. Other available Vi polysaccharide vaccines include Typherix (manufactured by GlaxoSmithKline [GSK]) and Typbar (manufactured by Bharat Biotech) [17].

Ty21a and Vi polysaccharide vaccines have limitations such as T-cell–independent immune response, hence, it is poorly immunogenic in young children; no booster response; and the need for repeat dosing. For the polysaccharide vaccines, these limitations can be overcome by conjugation of the Vi polysaccharide to a carrier protein. Conjugation of the polysaccharide to a carrier protein converts the immune response to be T-cell dependent, characterized by affinity maturation, subclass switching, and induction of memory [20]. Many TCVs are under development, and 3 have been licensed in India.

### Prototype Conjugate Vaccine: Vi-rEPA

Scientists at the US National Institute of Child Health and Disease developed the conjugation method that include the heterobifunctional cross-linking reagent N-succinimidyl-3-(2- pyridyldithio)-propionate or adipic acid dihydrazide as a linker to bind Vi to proteins. Using a nontoxic recombinant protein that is antigenically identical to *Pseudomonas aeruginosa* exotoxin A as a carrier protein, the resultant conjugates (Vi-rEPA) were more immunogenic in mice and juvenile Rhesus monkeys than the Vi alone [21]. In contrast to the T-independent properties of the Vi alone, conjugates of this polysaccharide with several medically relevant proteins induced booster responses in mice and juvenile Rhesus monkeys. This synthetic scheme was reproducible, provided high yields of Vi-protein conjugates, and was applicable to several medically relevant proteins such as diphtheria and tetanus toxoids [22]. The safety and immunogenicity of 2 investigational Vi-rEPA vaccines were evaluated in adults, 5- to 14-year-old children, and 2- to 4-year-old children in Vietnam. None of the recipients experienced a fever >38.5°C or significant local reactions after receiving an injection [23].

One or 2 doses of Vi-rEPA were evaluated in children aged 2 to 4 years. Six weeks after 1 dose, there was a 406-fold rise of immunoglobulin (Ig) G anti-Vi. At 26 weeks, IgG anti-Vi levels elicited by 2 injections of Vi-rEPA were higher than those elicited by only 1 injection (30.6 vs 20.4). Most importantly, IgG anti-Vi levels elicited by 2 injections of Vi-rEPA in children aged 2 to 4 years were higher than those elicited by Vi polysaccharide (alone) in children aged 5 to 14 years (30.6 vs 13.4; *P* = .01) [18]. The Vi-rEPA conjugate vaccine enhanced the immunogenicity of Vi alone and gave it T-cell–dependent properties. Vi-rEPA elicited a booster response in children aged 2 to 4 years whose levels of IgG Vi antibody were approximately 3 times as high as those elicited by Vi (alone) in children aged 5 to 14 years [18].

A double-blind, placebo-controlled, randomized efficacy study was conducted in children aged 2 to 5 years in Vietnam. A total of 11 091 children were injected twice, 6 weeks apart, with the Vi conjugate vaccine or saline. The overall efficacy after 27 months of active surveillance followed by 19 months of passive surveillance was 89% [24]. In a randomized, vaccine-controlled study of infants in Vietnam, Vi-rEPA was safe, elicited protective levels of IgG anti-Vi, and was compatible with Expanded Program on Immunization (EPI) vaccines. In this study, the enrolled newborns were randomized to receive vaccines of the EPI alone, with Vi-rEPA, or with *Haemophilus influenza* type b–tetanus toxoid conjugate at age 2, 4, and 6 months. There were no significant differences between the 3 groups in terms of safety. Of the infants vaccinated with Vi-rEPA, 95% had ≥3.5 ELISA unit (EU) at the end of 13 months after the fourth injection [25].

The US National Institutes of Health (NIH) has transferred the technology to the Lanzhou Institute of Biological Products (LIBP), which is part of the China National Biologics Group. LIBP conducted additional trials and submitted for in-country licensure in China in 2013 [17].

### Typbar-TCV: Bharat Biotech

Bharat Biotech in Hyderabad, India, developed a TCV using tetanus toxoid as the carrier protein with Vi polysaccharide. Typbar-TCV consists of 25 µg of Vi polysaccharide from *S*. Typhi conjugated to tetanus toxoid carrier protein in isotonic saline, licensed as a single intramuscular dose for use from age ≥6 months to 45 years. This vaccine was tested in children (aged 2 to 17 years) for safety, immunogenicity, and dose ranging. There was no significant difference between 2 doses of 25 µg and 2 doses of 15 µg [17].

In a clinical trial, the immunogenicity of Vi-TT was compared to that of the polysaccharide vaccine in 981 participants (age 6 months to 45 years). The investigators found 4-fold seroconversion rates in each treatment arm at 6 weeks post-vaccination. In a randomized, controlled trial, Typbar-TCV recipients attained higher anti-Vi IgG geometric mean titers (GMTs) 42 days after immunization (seroconversion [SCN], 97%; GMT, 1293 [95% confidence interval [CI], 1153–1449]) than recipients of Typbar (SCN, 93%; GMT, 411 [95% CI, 359–471]; *P* < .001). Typbar-TCV was highly immunogenic in the open-label trial (SCN, 98%; GMT, 1937 [95% CI, 1785–2103]). In a randomized, controlled trial, 2 years after vaccination, anti-Vi titers remained higher in Typbar-TCV recipients (GMT, 82 [95% CI, 73– 92]) and exhibited higher avidity (geometric mean avidity index [GMAI], 60%) than in Typbar recipients (GMT, 46 [95% CI, 40–53]; GMAI, 46%; *P* < .001). Typbar-TCV recipients achieved GMT of 48 (95% CI, 42–55) and GMAI of 57%. Typbar-TCV induced multiple IgG subclasses and strong booster responses in all ages. No serious vaccine-attributable adverse events were observed [26]. Based on these results, Bharat Biotech received marketing authorization for Typbar-TCV in India in 2013. Additional studies were conducted with Typbar-TCV to demonstrate noninterference of measles-containing vaccine when administered simultaneously to age-eligible recipients. Study results showed that TCV can be successfully coadministered with measles vaccine at age 9 months without interfering with the immune response to measles at 4 and 8 weeks post-vaccination compared to baseline [17]

The efficacy of the Typbar-TCV vaccine was assessed recently in an observer–participant-blinded study that used an established controlled human typhoid infection model in naive adult volunteers (aged 18 to 60 years; n = 103) in a nonendemic setting (the United Kingdom). Participants were randomized to receive a single parenteral dose of Typbar-TCV, Vi-PS (Typhim Vi, Sanofi-Pasteur), or a control (group ACWY meningococcal conjugate) vaccine. Both Vi vaccines contained 25 µg of Vi-polysaccharide per 0.5 mL dose [27, 28]. Approximately 1 month post-vaccination, participants were orally challenged with 1–5 × 10^4^ colony-forming units of *S*. Typhi Quailes strain (a wild-type strain originally isolated from a chronic carrier in Baltimore, Maryland), preceded by the ingestion of 120 mL of sodium bicarbonate buffer [29]. Different vaccine efficacy estimates of Typbar-TCV were obtained using clinical or microbiological diagnostic endpoints. Vaccine efficacy was estimated as 87.1% (95% CI, 47.2, 96.9) against a persistent fever (defined as fever ≥38˚C persisting for >12 hours) followed by positive blood culture for *S*. Typhi (Vi-TT attack rate 5% vs control attack rate 42%) compared to vaccine effectiveness of 52.3% (95% CI −4.2, 78.2) for the Vi-PS against the same endpoint (an attack rate of 20%). Effectiveness reported against bacteremia was 37.2% (95% CI, 11.8–64.7), while reported effectiveness was 89.5% (95% CI, 20.8, 98.6) against the typhoid triad (fever ≥38.0 °C plus headache and abdominal pain) [11]. Seroconversion was 100% in Typbar-TCV recipients and 88.6% in Vi-PS recipients, with significantly higher GMTs detected 1 month post-vaccination in Typbar-TCV vaccinees (GMT, 562.9 EU/mL [396.9, 798.4] vs 140.5 EU/ml [91.0, 216.9]; *P* < .001). An inverse straight-line relationship was demonstrable between the level of anti-Vi IgG titer and the probability of developing serologically defined typhoid but with no apparent antibody titer threshold. Overall, Typbar-TCV induced satisfactory antibody response and memory, where higher levels of anti-Vi antibody correlated with increased protection [27, 28]. WHO prequalification was awarded to Bharat Biotech for Typbar-TCV in January 2018. 

### PedaTyph: BioMed

PedaTyph was the first TCV to be licensed in India. It consists of 5 µg of Vi polysaccharide from *S.* Typhi conjugated to 5 μg of tetanus toxoid protein in isotonic saline. A randomized comparative trial was conducted in 400 healthy Indian children aged 3 months to 5 years who received 1 dose of PedaTyph (n = 200) or 2 doses 8 weeks apart (n = 200). In 101 children aged <2 years and 24 children aged <1 year who were available for follow-up, a seroconversion rate (≥4-fold increase over preimmunization titer) of 83% was reported at 8 weeks post-vaccination, with the highest seroconversion rate in infants (seroconversion rates of 73%, 89%, and 96% for children aged >2 years, ≤2years, and <1 year, respectively) [30]. In a follow-up of the first study cohort of 400 children, 40 children who received either 1 or 2 doses of PedaTyph were recalled 30 months after vaccination to assess the longevity of immune response [31]. Anti-Vi IgG titers were reported to be significantly higher in vaccinated children (1 dose or 2 doses) at 30 months post-vaccination compared to nonvaccinated children, and the titers in the 2-dose group were reported to be higher than in the single-dose group but not significantly [31]. A quasi-randomized, open-label trial was conducted post-licensure in 905 Kolkata children aged 6 months to 12 years who received 2 doses of PedaTyph 6 weeks apart and were followed with active surveillance (weekly telephone calls plus monthly school visits) for 1 year, along with 860 unvaccinated controls [32]. Incidence of culture-positive typhoid fever in the control group was 1.27% and zero in the vaccinated group. In a subgroup evaluated for immunogenicity, an antibody titer value of 1.8 EU/mL (95% CI, 1.5, 2.2), 32 EU/mL (95% CI, 27.0, 39.0), and 14 EU/mL (95% CI, 12.0,17.0) at baseline, 6 weeks, and 12 months, respectively, was observed. Seroconversion among the subgroup was 100% after 6 weeks post-vaccination and 83% after 12 months considering a 4-fold rise from baseline. The efficacy of the vaccine was 100% (95% CI, 97.6, 100) in the first year of follow-up, with minimal adverse events post-vaccination [32]. PedaTyph was licensed in India in 2008 and is recommended for children aged >3 months as a single dose of 0.5 mL followed by boosters at age 2.5 to 3 years [11].

### Vi-TT: Zydus Cadila

Zydus Cadila developed a TCV using tetanus toxoid as the carrier protein. Phase 2 and 3 clinical trials were conducted at 7 sites in India for immune noninferiority with Typbar-TCV (238 participants in all age groups). After this noninferiority study, a dossier was submitted to Indian National Regulatory Authority for marketing authorization in India. This vaccine is now licensed in India as a single dose of 25 µg from age 6 months onward [17].

### Vi-CRM_197_: GVGH

Vi-CRM_197_, developed by GSK Vaccines Institute for Global Health (GVGH), includes CRM_197_ as the carrier protein, which is a nontoxic mutant of diphtheria toxin. In phase 1 and 2 trials, safety and immunogenicity of Vi-CRM_197_ vaccine against *S*. Typhi were tested in European adults. Vi-CRM_197_ was found to be safe and as immunogenic as Vi-PS [33]. A phase 2 trial with 320 participants including adults, children, and infant was conducted in India, Pakistan, and the Philippines. The study concluded that Vi-CRM_197_ was safe and immunogenic in endemic populations of all ages, although the responses were short-lived. Given at age 9 months concomitantly with the measles vaccine, Vi-CRM_197_ showed promise for potential inclusion in EPI schedules in countries endemic for typhoid [34]. GVGH has modified the technology, and it has been transferred to Biological E and is in full clinical development [17].

### Vi-DT: IVI

With initial know-how from the US NIH, IVI scientists developed the TCV, which consists of the Vi polysaccharide purified from *S.* Typhi chemically conjugated to diphtheria toxoid. IVI transferred the technology for production and quality control of Vi-DT to 3 manufacturing partners (SK Chemicals, South Korea; Biofarma, Indonesia; and Incepta, Bangladesh) and is working with them to complete the clinical development with the aim of local licensure and WHO prequalification. Two partner manufacturers (SK Chemicals, South Korea, and Biofarma, Indonesia) have completed phase 1 clinical trials. Phase 2 clinical trials by both the manufacturers are currently ongoing.

## DISCUSSION

It should be noted that these vaccines are designed to protect against disease due to *S*. Typhi. To the extent that *S.* Paratyphi A contributes to the overall cases of typhoid fever in a given region, there will be a background level of clinical enteric fever that will not be affected by the conjugate vaccines. In addition, we need to understand the difficulty in defining criteria for protection in vaccine studies. Various criteria can and have been used to define efficacy: overall clinical disease, fever, duration of fever, bacteremia, and others. For large-scale vaccination efforts and follow-up, efficacy will need to be precisely defined. A standard field definition of typhoid fever should be considered, such as fever 38.0°C or higher followed by bacteremia. Future research on whether conjugate vaccines have any effect on enteric infection and shedding is also warranted. This issue is of great importance in any large-scale vaccination effort.

## CONCLUSIONS

**Figure 1.**
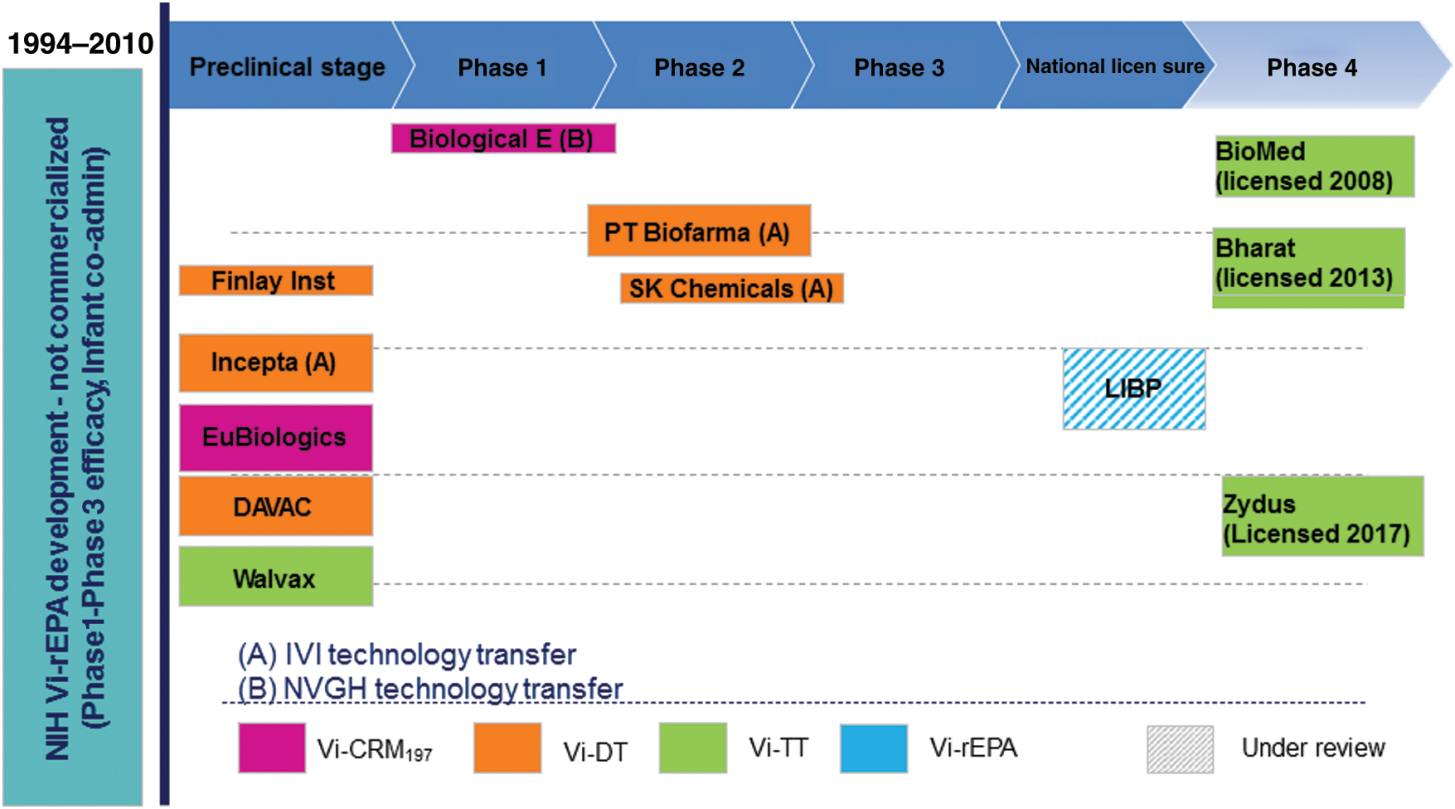
The typhoid conjugate vaccine pipeline. Abbreviations: LIBP, Lanzhou Institute of Biological Products; NIH, US National Institutes of Health.

The current TCV pipeline (Figure 1) is robust and ensures the availability of many TCVs in the near future to address the unmet public health need and market demand.

## Notes

***Supplement sponsorship.*** This supplement was sponsored by Coalition against Typhoid, Sabin Vaccine Institute, Bill & Melinda Gates Foundation.

***Potential conflicts of interest.*** T. S. and S. S. are employed by the Internation Vaccine Institute and are involved in SK Bioscience, South Korea, and Biofarma, Indonesia, Vi-DT vaccine development projects. Both authors have submitted the ICMJE Form for Disclosure of Potential Conflicts of Interest. Conflicts that the editors consider relevant to the content of the manuscript have been disclosed.
